# The air–water interfacial nitrogen cycle produces irrigatable-level ammonium nitrate

**DOI:** 10.1039/d5sc05754j

**Published:** 2025-10-14

**Authors:** Xiaowei Song, Chanbasha Basheer, Jinheng Xu, Richard N. Zare

**Affiliations:** a Department of Chemistry, Stanford University CA 94305 USA zare@stanford.edu; b Chemistry Department, King Fahd University of Petroleum and Minerals Dhahran 31261 Saudi Arabia cbasheer@kfupm.edu.sa

## Abstract

We report a sustainable, air-based strategy for synthesizing ammonium nitrate (NH_4_NO_3_) by harnessing the redox-active properties of microscale air–water interfaces. The process proceeds through two sequential reactions: (1) the nitrogen oxidation reaction (NOR), generating nitrate (NO_3_^−^) from atmospheric N_2_, and (2) the nitrate reduction reaction (NO_3_RR), converting nitrate into ammonium ions (NH_4_^+^). In the first step, ambient air is introduced into a recirculating microbubble system, where solar irradiation and a water-soluble photocatalyst drive the efficient oxidation of N_2_ to nitrate, producing NO_3_^−^ at a rate of 500 μmol L^−1^ h^−1^. In the second step, atomized water microdroplets are sprayed across a Fe_3_O_4_–Nafion–CuO mesh, generating an extensive air–water interfacial area that promotes the reduction of nitrate and nitric oxide intermediates to NH_4_^+^. Over 12 hours, this tandem process yields a 50 mL aqueous solution containing 0.94 mM NH_4_NO_3_ and 4.42 mM HNO_3_, derived entirely from air and water. This carbon-free and catalyst-assisted platform offers a decentralized and environmentally friendly approach to nitrogen fixation, with immediate applications in hydroponic systems, where controlled nutrient delivery is essential. The integration of solar photocatalysis with microdroplet interfacial chemistry establishes a viable foundation for next-generation green fertilizer technologies.

## Abstract 2

We report a sustainable, air-based strategy for synthesizing ammonium nitrate (NH_4_NO_3_) by harnessing the redox-active properties of microscale air–water interfaces.

## Graphical abstract

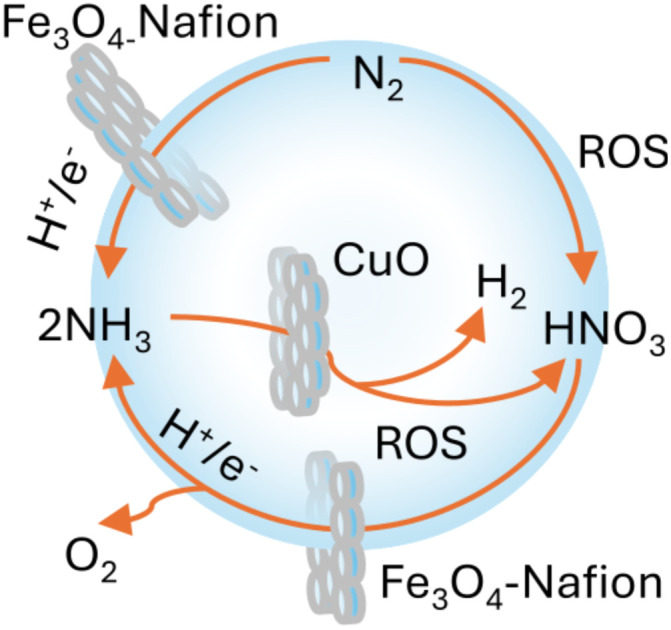

## Introduction

Ammonia plays a fundamental role in building life and supporting modern human society. It is one of the most important fertilizers for providing nitrogen sources, which is indispensable for basic life building blocks such as amino acids and nucleotides. With the invention of the Haber–Bosch process (HBP) for industrial ammonia synthesis in the early 20th century, world food production surged sharply, accompanied by a significant increase in human population.^1,2^ Unfortunately, this classical ammonia production method begins with nitrogen and hydrogen derived from the steam reforming of methane gas. The harsh conditions (15–25 MPa and 400–500 °C) require an intensive energy input and account for approximately 1–2% of global energy consumption.^3,4^ HBP is also responsible for a substantial portion of industrial greenhouse gas emissions, given that it often relies on fossil fuels, particularly natural gas, for both hydrogen production and as an energy source.^5^ Efforts to reduce the carbon footprint of HBP include exploring alternative hydrogen sources and efficient catalytic strategies for the nitrogen reduction reaction (NRR) at lower temperatures and pressures.^6^

Water is the ideal hydrogen resource compared to methane because it is carbon-free, clean, widely available, and renewable in nature. Compared to bulk water, numerous studies have demonstrated that micron-sized droplets can serve as highly active reactors for both oxidation and reduction.^7–15^ It can not only be a source of reducing species but also a source of reactive oxygen species, which have also been observed across the air–water interface of microdroplets in many showcase experiments.^16–21^ Apart from water microdroplets, some research study also observed the generation of hydrogen peroxide (H_2_O_2_) and hydroxyl radicals (OH˙) at another reversed form of air–water interface of electrogenerated microbubbles on an electrode surface.^22,23^ Regardless of whether it is microdroplets or microbubbles, the large gas-contact area and redox potential of the air–water interface region provide a promising 2D environment for nitrogen fixation.

Previously, we successfully identified the formation of ammonia by spraying N_2_ with water microdroplets through a copper oxide (CuO) mesh coated with a catalyst (Fe_3_O_4_–Nafion). The ammonia generated can reach a concentration of 60 μM within 1 hour of operation under ambient conditions.^24^ Later, a more efficient device was developed to conduct on-site ammonia synthesis using water vapor and dinitrogen in the air as the feedstock. The ammonia concentration increased to 270.2 ± 25.1 μM after 2 hours of operation, which is sufficient for some agricultural irrigation purposes.^25^ We also detected NO_3_^−^ generation during the microdroplet spraying process. The air–water interfacial hydroxyl radicals lead to the oxidation of either N_2_ ^26–29^ or the generated NH_3_.^30,31^ This observation further supported the fact that the water microdroplet interface may have an asymmetric redox ability to conduct both nitrogen reduction and oxidation, which formed ammonium nitrate (NH_4_NO_3_) as the actual product during our previous ammonia synthesis from the nitrogen in air.

Inspired by the intermolecular conversion cycle composed of N_2_, NO_3_^−^, and NH_4_^+^, we are motivated to take advantage of the redox process of the micron-sized air–water interfacial environment for nitrogen fixation. A sequential process was conducted including the first step of the nitrogen oxidation reaction (NOR) that converts N_2_ to NO_3_^−^ in a circulating microbubble system, followed by the nitrate reduction reaction (NO_3_RR) that partially converts the NO_3_^−^ into NH_4_^+^ in a spraying microdroplet system, resulting in NH_4_NO_3_ and extra HNO_3_ as final aqueous products.

## Results

### Highly efficient oxidation of nitrogen by microbubbles of air in water

**Fig. 1:**
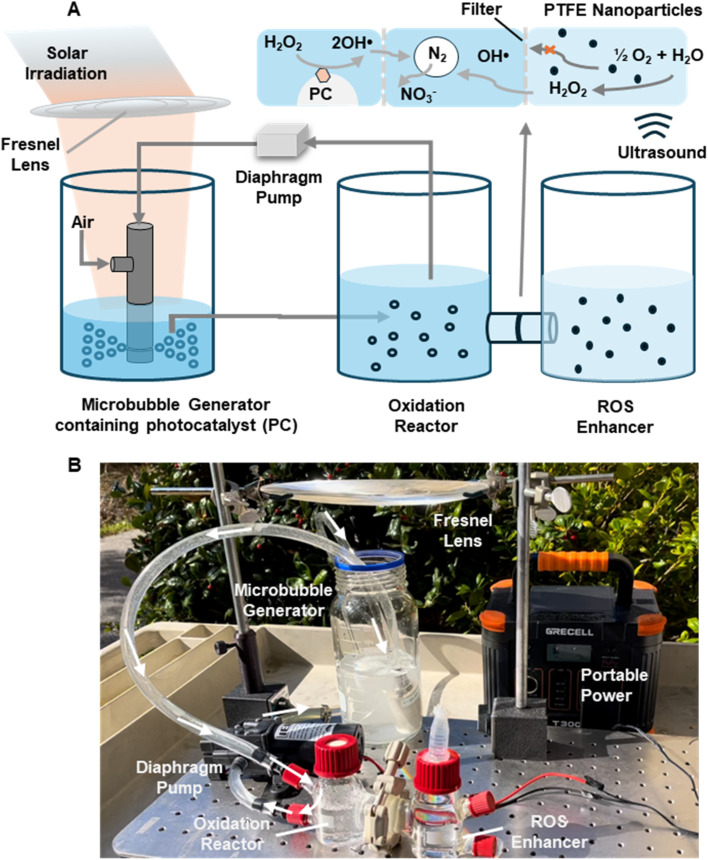
A continuous microbubble system for nitrogen oxidation to nitrate. (A) Schematic illustration of the setup, which consists of a microbubble generator, an oxidation reactor, and an additional ROS enhancer. (B) Photograph of the real microbubble setup working outdoors, driven by a portable power supply under natural sunlight irradiation.

The first step of our method is to oxidize the nitrogen in the air into nitrate. We developed a dinitrogen-to-nitrate reactor assembly to achieve this goal. This device mainly consists of a microbubble generator, a nitrogen oxidation reactor, and a reactive oxygen species (ROS) enhancer (Fig. 1). The microbubble generator can effectively increase the surface-to-volume ratio of the bulk water and contacting area of the air–water interface, which has been proven to be a highly reactive region rich in ROS, such as hydroxyl radical (OH˙), superoxide anion (O_2_˙^−^), and hydrogen peroxide (H_2_O_2_).^32,33^ Nitrogen in the air can be fully dispersed into bulk water to maximize the exposure of nitrogen to the active interfacial region and extend the oxidation period.

To further enhance the efficiency of the nitrogen oxidation process, an optional ROS enhancer can be coupled with a microbubble generator and an oxidation reactor. The generated microbubbles can be transported to the oxidation reactor to fully interact with the H_2_O_2_ generated from the reactive oxygen species (ROS) enhancer unit. The ROS enhancer is filled with deionized water with polytetrafluoroethylene (PTFE) nanoparticles dispersed inside. An additional amount of 300 μM H_2_O_2_ can be produced by ultrasonicating the enhancer unit for 1 hour in advance before running the microbubble generator.^34,35^ This extra 300 μM H_2_O_2_ can freely diffuse into the neighboring oxidation reactor and reach an equilibrium within 2 hours (Fig. S1), leading to the enhancement of the nitrogen to nitrate conversion across the interfacial region. The ultrafiltration film embedded between the oxidation unit and enhancer unit can block the PTFE particles from diffusion and clogging in the diaphragm pump and narrow channel within the microbubble generator.

**Fig. 2:**
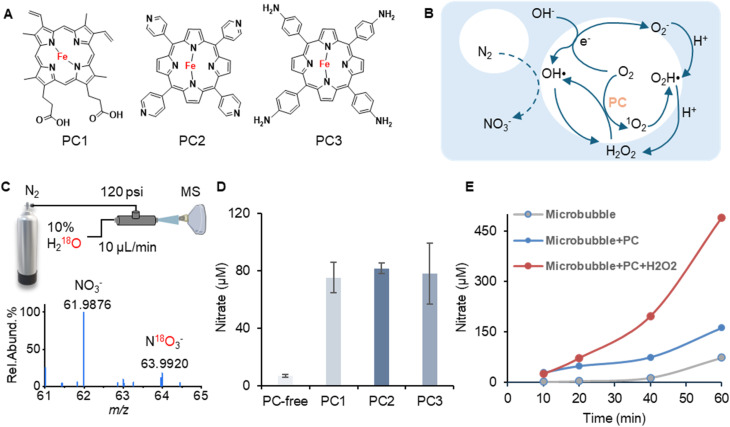
Investigating nitrogen oxidation and final nitrate production facilitated by the photocatalyst and externally generated H_2_O_2_ by contact-electrocatalysis. (A) Chemical structures of three different photocatalysts (PC); (B) diagram of the reactive oxygen species interconversion facilitated by PC and the air–water interface; (C) high-resolution mass spectrometry confirmation of the hydroxyl radical-involved nitrate generation by using native water spiked with 10% ^18^O-labelled water; (D) the improvement of nitrate production by adding different types of photocatalysts in the microbubble system; (E) the improvement of nitrate production with time by adding H_2_O_2_ externally generated from the ROS enhancer unit.

The OH˙ around the air–water interface can be formed through multiple ways by interacting with the surrounding air. First, the OH˙ can directly come from OH^−^ by losing one electron, which is thermodynamically favorable across the air–water interface based on the theoretical calculation.^36^ Second, the OH˙ can be indirectly generated from H_2_O_2_ in the presence of oxygen (O_2_), protons, and solvated electrons. It has been discovered that air–water interfaces can not only induce the generation of O_2_^−^, hydroperoxyl radical (OOH˙), and singlet oxygen (^1^O_2_) but also promote the photocatalysis process.^37–41^ In this study, three water-soluble iron porphyrins were also investigated in the microbubble circulating system (Fig. 2A). Iron porphyrins can serve as photocatalysts (PCs) to break H_2_O_2_ into active OH˙ under solar light irradiation. Singlet oxygen can also be generated by the photocatalyst upon light excitation. All these species can promote the OH˙ formation by interacting with the water molecules through different pathways.

As shown in Fig. 2C, during a 30-minute operation, the average concentration (*n* = 3) of nitrate increases from 6.7 μM to 74.9 μM (PC1), 81.5 μM (PC2), and 77.9 μM (PC3) with the addition of PC in the microbubble system, respectively. The device can achieve an even better performance when using the ROS enhancer. Within 1 hour of operation, the nitrate concentration can reach 489.5 μM with the use of the extra ROS enhancer plus PC2 group compared to 162.1 μM for the PC2 group and 73.9 μM for the microbubble alone group (Fig. 2D).

### Air–water interfacial nitrogen cycle

Our previous studies have already demonstrated that ammonia can be formed by spraying water microdroplets and dinitrogen through the catalyst-coated mesh (Fe_3_O_4_–Nafion@CuO).^24,25^ Both ammonia and dinitrogen gas can also be directly oxidized into nitrates by spraying microdroplets.^26–31^ These facts indicate an air–water interfacial nitrogen cycle composed of dinitrogen, ammonia, and nitrate species.

**Fig. 3:**
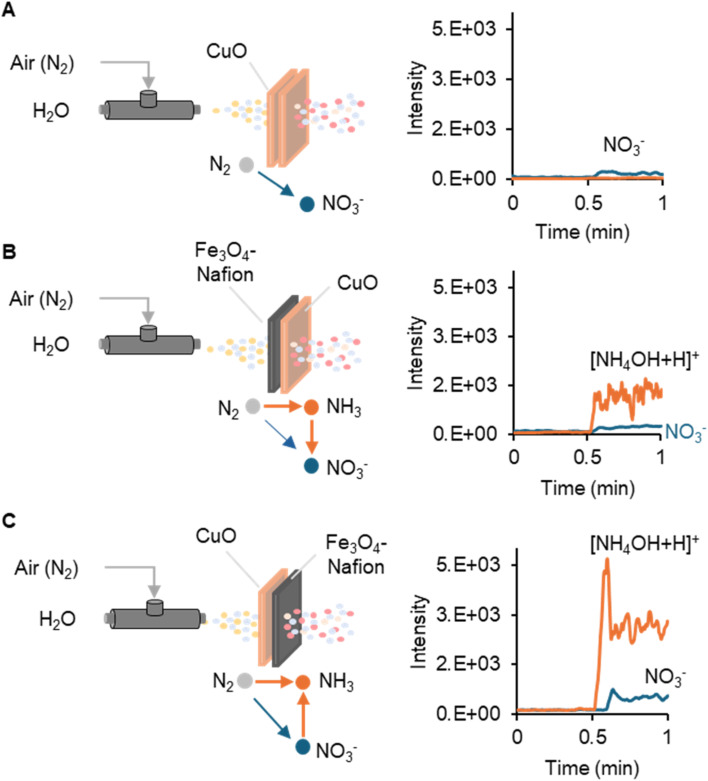
Online mass spectrometry monitoring nitrogen oxidation and reduction products after spraying water microdroplets and air through different types of metal oxide meshes: (A) double layers of CuO meshes; (B) Fe_3_O_4_–Nafion-coated CuO mesh in the front and another CuO mesh in the back; (C) CuO mesh in the front and Fe_3_O_4_–Nafion-coated CuO mesh in the back.

Because nitrate reduction into ammonia is a more effective way requiring less energy-cost compared to direct reduction of dinitrogen gas,^42–44^ we are motivated to explore the new path of ammonia production further, starting from nitrate. We use a classical coaxial capillary atomizer to generate a water microdroplet spray with the aid of compressed air (100 psi) as the nebulizing gas (Fig. 3). A linear ion trap mass spectrometry was employed to monitor the nitrogen species and their changes.

When we let spraying water microdroplets and air pass through the CuO mesh, the ion intensity of *m*/*z* 62 (NO_3_^−^) was doubled from 7.2 × 10^1^ to 1.8 × 10^2^ (Fig. 3A). This phenomenon indicates the enhancement of nitrogen oxidation with the involvement of a CuO mesh, which serves as a Fenton-like catalyst.^30^ When another Fe_3_O_4_–Nafion@CuO mesh was stacked in front of the CuO mesh for interacting with the microdroplet spray, the ion intensity of *m*/*z* 36 had a sharp surge from 6.8 × 10^1^ to 1.5 × 10^3^, indicating the generation of ammonia ([NH_4_OH + H]^+^) from dinitrogen gas. Meanwhile, the NO_3_^−^ ion at *m*/*z* 62 also reached a level of 2.3 × 10^2^ (Fig. 3B), which is slightly higher than what we observed when spraying through the CuO mesh alone. This is because although both N_2_ and NH_3_ can contribute to NO_3_^−^ formation, NH_3_ is more readily oxidized than N_2_. Finally, when we reversed the Fe_3_O_4_–Nafion/CuO (back) and CuO (front) meshes, the ion intensity of *m*/*z* 36, representing [NH_4_OH + H]^+^, increased to 2.9 × 10^3^, compared to the previous value of 1.5 × 10^3^ (Fig. 3C). This result supports our hypothesis that converting N_2_ to NO_3_^−^ as the first step can effectively improve the subsequent nitrogen reduction into NH_3_. It is worth noting that the ion intensity of *m*/*z* 62 representing NO_3_^−^ also further increased to 5.1 × 10^2^ compared to the previous 2.3 × 10^2^ level. This may suggest that NO_3_^−^ is also a possible byproduct from unavoidable NH_3_ oxidation.

### Critical nitrogen species showing the ongoing NO3RR to form ammonia

**Fig. 4:**
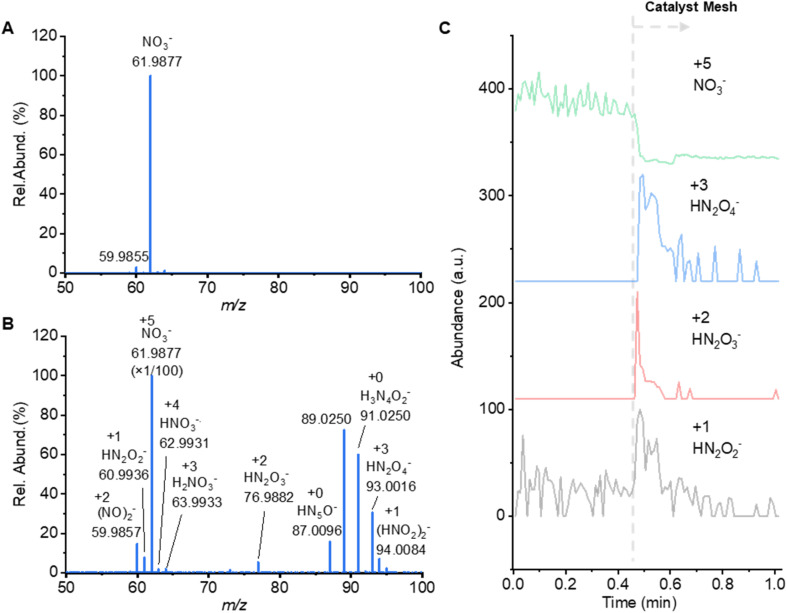
Characterizing valence changes of the nitrogen element from stepwise hydrogenation during the nitrate reduction process. (A) The mass spectrum was collected in negative mode by spraying water microdroplets containing nitrate sodium directly into the mass spectrometer. (B) The mass spectrum was acquired by spraying water microdroplets containing sodium nitrate through the CuO mesh coated with the Fe_3_O_4_–Nafion catalyst. (C) Extracted ion chromatograms of nitrate and representative NO_*x*_^−^ species with reduced valence.

An Orbitrap high-resolution mass spectrometer (HRMS) was employed to gain a deep insight into the stepwise NORR process in the negative scanning mode. When directly spraying the air and water, a strong peak at *m*/*z* 61.9877 (NO_3_^−^) can be detected (Fig. 4A). When the Fe_3_O_4_–Nafion@CuO mesh was mounted in the middle of the nozzle atomizer and the MS inlet, a set of associated peaks appeared in the mass spectrum. Exploring elemental compositions, they can be putatively annotated as various N_*x*_O_*y*_H_*z*_ species. Although the detailed molecular structure cannot be elucidated by the HRMS alone, the elemental composition information is sufficient to clarify the valence profile of these N_*x*_O_*y*_H_*z*_ species, which varied from +5 (*m*/*z* 61.9877, NO_3_^−^) to +4 (*m*/*z* 62.9931, HNO_3_˙^−^), +3 (*m*/*z* 63.9933, H_2_NO_3_^−^, and *m*/*z* 93.0016, HN_2_O_4_^−^), +2 (*m*/*z* 76.9882, HN_2_O_3_^−^), +1 (*m*/*z* 61.0040, HN_2_O_2_^−^) (Fig. 4B). Although the NO_3_^−^ peak at *m*/*z* 61.9877 remained the base peak, its signal strength immediately dropped by 80% (Fig. 4C). In contrast, these N_*x*_O_*y*_H_*z*_^−^ species were only present and appeared when the catalyst mesh was mounted in front of the spray. The apparent valence changes from +5 to +1 indicate the ongoing stepwise hydrogenation and reduction processes.

### Theoretical calculation for ammonia synthesis from the NO_3_RR

**Fig. 5:**
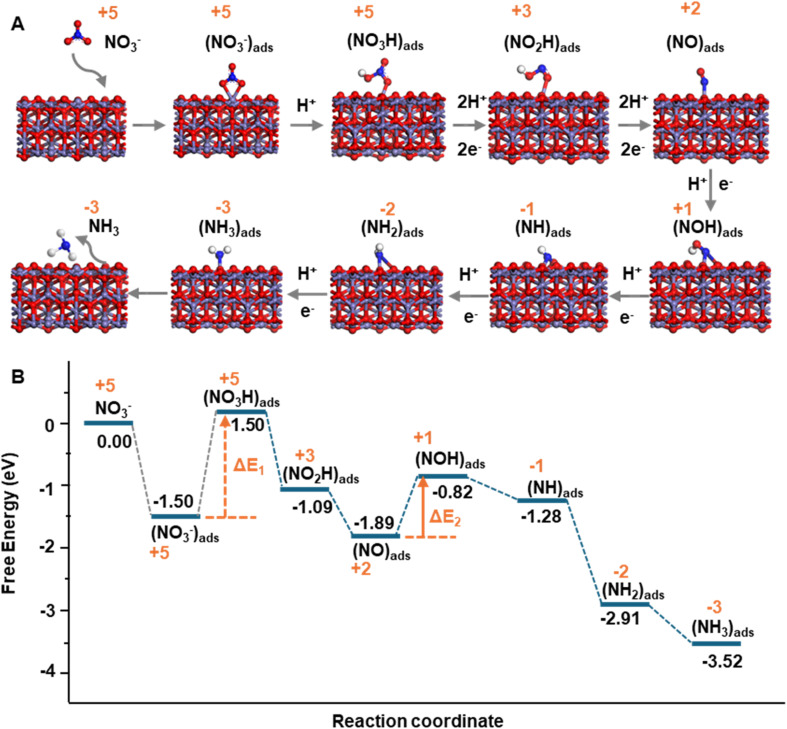
DFT calculation of nitrate reduction into ammonia. (A) Diagram of the nitrate molecule absorption and stepwise hydrogenation across the Fe_3_O_4_ surface; (B) energy changes during the whole nitrate reduction process.

We further conducted theoretical computations on the stepwise interaction between the catalytic center and the N_*x*_O_*y*_H_*z*_^−^ species, molecular structure changes, and the corresponding potential energy landscape across the entire NO3RR process using density functional theory (DFT) calculations. The crystal face of (311) of Fe_3_O_4_ was selected for nitrate absorption and activation because it was confirmed to be the major form by X-ray diffraction (XRD) (Fig. S3). Throughout the interaction diagram shown in Fig. 5A, the change of nitrogen valence from +5 to +1 is consistent with the captured N_*x*_O_*y*_H_*z*_^−^ species in the HRMS experiment.

Regarding the potential energy change, after one NO_3_^−^ molecule was chemically absorbed to the ferrous center by forming two Fe–O coordination bonds, one Fe–O bond will be liberated by adding a H^+^ donated from water microdroplets. This is the first rate-limiting step with the highest activation energy (3 eV) across the whole process because one of the Fe–O bonds needs to be liberated to make the oxygen available to accept the donated proton. Thereafter, stepwise hydrogenation can spontaneously proceed without overcoming any energy barrier until the step of (NO)_ads_ reduction into (HNO)_ads_. This is the second rate-limiting step with a relatively high energy barrier around 1.07 eV, and all the other steps are energetically downhill (Fig. 5B).

Previous studies have demonstrated that spraying water microdroplets can not only cause contact electrification at the water–metal mesh interface^45–48^ but also induce microlightning during droplet splitting.^49^ The microlightning of microdroplets can ionize the surrounding molecules, even those relatively inert molecules such as xenon (ionization energy around 12 eV). The negative contact electrified potential between microdroplets and catalyst mesh can provide the driving force to overcome the energy barriers of the two steps. The contact electrified potential was also attributed to the use of Nafion whose hydrophobic perfluoroalkyl backbone can accept, stabilize, and facilitate reducing electrons to transfer from the microdroplet surface to the N_*x*_O_*y*_H_*z*_^−^ species. The sulfonic acid group in the Nafion film can also facilitate the reduction by H^+^ transfer. Our previous HRMS test successfully captured the *m*/*z* 62.9931 (HNO_3_^−^) and *m*/*z* 61.0040 (HN_2_O_2_^−^) ions, which represent the nitrogen valences of +5 and +1, respectively. This provides evidence that these two critical steps can be achieved in the microdroplet interfacial catalysis process.

### Highly efficient nitrate reaction to ammonia at the air–water interface

**Fig. 6:**
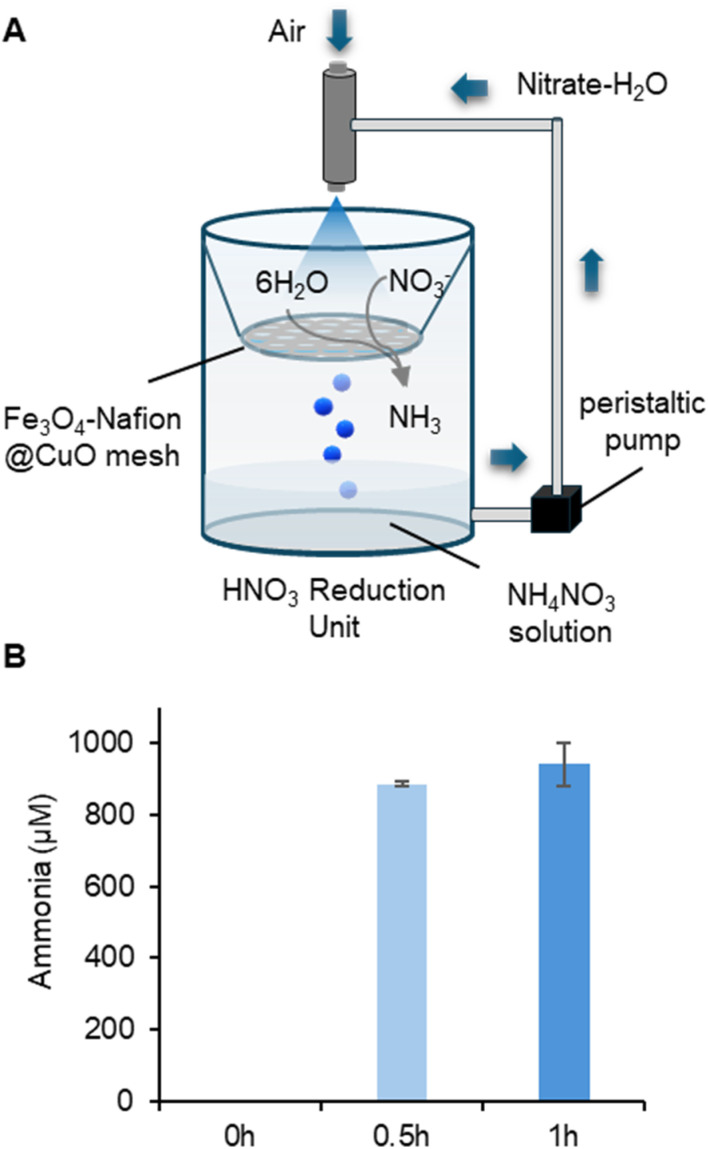
Ammonia production utilizes nitrate as the starting material. (A) Diagram of the scaled-up setup equipped with a 100 mL volume reactor embedded with the Fe_3_O_4_–Nafion@CuO catalyst mesh, a nozzle atomizer for continuous spraying, and a peristaltic pump for water solution circulation. (B) The mM level of ammonia production with a 1-hour operation feed with a nitrate solution.

After achieving the oxidation of N_2_ to NO_3_^−^ in microbubbles and reduction of NO_3_^−^ to NH_3_ in microdroplets, we combined these two steps sequentially to maximize the ammonium nitrate production. We first ran the microbubble reactor described above for 12 hours to produce approximately 6.3 mM nitrate in 50 mL solution. Then, the 50 mL nitrate solution was transferred into a scaled-up nozzle atomizer embedded with the Fe_3_O_4_–Nafion@CuO catalyst mesh, a nozzle atomizer for continuous spraying, and a peristaltic pump for water solution circulation (Fig. 6A). Finally, 0.94 mM ammonia can be produced within only 1 hour of running (Fig. 6B). Given the unreacted nitrate in the solution, there was 0.94 mM ammonium nitrate and an additional 4.42 mM nitrate produced in total over 13 hours of operation. This concentration is sufficient for direct irrigation purposes.^50,51^ Additionally, we found that the solution pH is a critical factor that affects the ammonia production efficiency. A pH of 1.0–2.0 can achieve near-mM level production, whereas a pH higher than 2.0 can only achieve a concentration of 147–188 μM ammonium nitrate (Fig. S4). Nonetheless, even the 147–188 μM ammonium nitrate plus the unreacted 6.2 mM nitrate is still suffice for the irrigation, regardless of whether nitrogen element is present in the form of ammonia or nitrate.

The stability of the Fe_3_O_4_–Nafion–CuO catalyst was first investigated by mounting in front of sprayed microdroplets and nebulizing the gas for 1 hour of continuous running. The produced ammonia ion intensity gradually drops to a relatively stable level after 30 minutes (Fig. S5A). Thereafter, the activity of the catalyst during the long-term storage was tested by successively using the same catalyst mesh at 1, 7, 14, 21, and 30 days after the preparation. As a result, the catalyst can retain the active performance for at least two weeks when stored at room temperature and in an open environment (Fig. S5B).

## Discussion

Our study has demonstrated how to more effectively utilize a micron-sized air–water interfacial environment to convert N_2_ into NH_4_NO_3_, a commonly used fertilizer in agriculture. By integrating two reactions into a closed-loop nitrogen system where atmospheric N_2_ is first oxidized to NO_3_^−^ and then reduced to NH_3_, the process effectively emulates natural nitrogen cycling. This tandem approach offers a sustainable pathway for decentralized fertilizer production, reduces greenhouse gas emissions, and aligns with the principles of green chemistry and a circular economy.

Compared to dinitrogen, NO_3_^−^ is a better alternative nitrogen source. Compared to the NRR, the NO_3_RR offers a cost-effective strategy (NO_3_^−^ + 6H_2_O + 8e^−^ → NH_3_ + 9OH^−^). First, the solubility of sodium nitrate (880 g L^−1^) is significantly higher than that of N_2_ in water (20 mg L^−1^), transforming the gas–liquid–solid tri-phase heterogeneous catalysis into a two-phase process. More importantly, the dissociation of single and double bonds between N and O in NO_3_^−^ needs far less energy (48.7 kcal mol^−1^) compared to the N≡N triple bond, which has an extremely strong bond dissociation energy (225 kcal mol^−1^).^52–55^ In terms of energy input, contact electrification and triboelectrification offer effective means of conducting interfacial redox reactions, capitalizing on renewable energy sources such as wind, tide, and water evaporation.^45–48^ The charge separation will occur across the interfaces of the gas, solid, and liquid phases, which possess different abilities in capturing electrons or protons, providing the electric potential difference that drives various chemical reactions.

The air interface region of microbubbles and microdroplets provides a redox-active microenvironment that enables the direct activation of ambient nitrogen and oxygen, producing ROS such as OH˙, O_2_^−^, and singlet oxygen (^1^O_2_).^37,38^ These air–water interfacial redox properties can be further enhanced through collaboration with photocatalysis and contact electrocatalysis. PTFE nanoparticles and a molecular iron-based photocatalyst were introduced into the reaction system to maximize the nitrate production from air in the first critical step. Ultrasonicating PTFE dispersion can be the pre-production step to provide extra H_2_O_2_ on the baseline level of ROS generated by microbubbles. Iron is an ideal metal to incorporate into a photocatalyst. First, it can absorb solar radiation to activate triplet oxygen into singlet status (^3^O_2_ → ^1^O_2_), which is highly reactive and can be converted to superoxide by capturing free electrons (^1^O_2_ + e^−^ → O_2_^−^). This process can be well coupled with the single electron transfer process occurring across the microbubble interfacial region (OH^−^ → OH˙ + e^−^). On the other hand, the iron-center porphyrin photocatalyst also allows a Fenton-like reaction, which can break the relatively stable H_2_O_2_ into more reactive OH˙ for N_2_ oxidation.^56–58^

Iron oxide-based catalysts have shown significant activity in electrocatalytic hydrogen evolution reactions (HERs), effectively promoting water splitting and generating active hydrogen species (*H), which are essential for the hydrogenation steps in the NO3RR.^57^ Compared to copper-based catalysts, iron oxides exhibit stronger affinity for nitrite (NO_2_^−^) intermediates, enhancing their further reduction to ammonia.^58^ A porous CuO mesh-supported Fe_3_O_4_–Nafion catalyst demonstrates improved NO_3_^−^ adsorption and electron transfer due to the synergistic interaction between Cu and Fe active sites, which weakens the N–O bonds and lowers the activation energy barrier. In this tandem system, Cu primarily facilitates the reduction of NO_3_^−^ to NO_2_^−^, while Fe_3_O_4_ catalyzes the subsequent hydrogenation to NH_3_. The introduction of a microdroplet interface further amplifies the process by providing a large electric field, as well as a high interfacial surface area, which enhances mass transport and concentrates NO_*x*_ intermediates and H˙ species. Overall, the combination of tandem catalysis and interfacial microdroplet dynamics enables efficient and selective nitrate-to-ammonia conversion under ambient aqueous conditions.

Ammonium nitrate (NH_4_NO_3_) is a commonly used nitrogen fertilizer that provides both NH_4_^+^ and NO_3_^−^, two essential forms of nitrogen that plants readily absorb. Its balanced application is critical for optimal plant development, as excess nitrate can contribute to environmental issues including groundwater contamination, whilst too much ammonium can induce osmotic stress and toxicity in sensitive crops.^50^ This is especially crucial in controlled situations such as hydroponics, where accurate nutrient management is required. A concentration range of 1 to 3 mM L^−1^ ammonium nitrate^51^ is typically suitable for hydroponic plant seedlings and other controlled hydroponic farming systems. This range ensures that the plants receive sufficient nitrogen without causing osmotic stress. Our current method of synthesis can reliably achieve this concentration range, demonstrating its effectiveness and feasibility. This means that the suggested method can be used to produce ammonium nitrate on-site, *in situ*, regularly, ensuring a sustainable supply. This makes it a valuable method for managing nutrients in hydroponic systems, providing both long-lasting and precise control.

The energy input is contributed by photon energy stemming from solar irradiation, electric energy for running a diaphragm pump, and kinetic energy released from the compressed gas in cylinder, generating microbubbles and microdroplets to create a more reactive air–water interface area. We conducted an estimation of these three sources of energy consumption within 12 hours of micro-bubbling stage and 1 hour of microdroplet spraying stage (Tables S1–S3). As a result, electronic energy only accounts for 6% of the total energy, and the kinetic energy stored from the compressed gas cylinder can be negligible (less than 1%). Because solar energy can be regarded as a free source, and kinetic energy can be negligible, the price of HNO_3_ and NH_3_ is calculated by dividing the cost of electricity with generated product weights. The calculation results show US $5.98 per gram HNO_3_ and US $126.4 per gram NH_3_ (Table S4). Compared to the ammonia's global market price around US $0.00047 per gram and nitric acid's price of approximately US $ 0.00038 per gram, we acknowledge that the cost of the present method is still not competitive with the HBP from the point of view of energy cost. Nonetheless, this method still provides the potential solution that has advantages in replacing fossil energy with carbon-free, clean energy from the point of view of environmental protection.

To further improve the production efficiency and lower the energy cost, the larger-scale bubbling system is still under investigation, which will be independent work in near future. First, a larger volume microbubble reactor should be introduced to increase the amount of dinitrogen gas to be oxidized. The interfacial nitrogen oxidation time can also be extended by raising the height of the bulk water surface. The microbubble density can be increased by replacing a single microbubble generator with a generator array. Dinitrogen molecules can be more effectively dispersed and oxidized by generating nano-sized bubbles. Given these measures, we believe that the produced nitrate can reach over hundreds of mM and even the M level at the gallon scale.

## Conclusion

We have demonstrated an air–water interfacial redox approach to produce mM-level ammonium nitrate under ambient conditions, reducing the requirement for energy-intensive processes involved in traditional ammonia and nitric acid production. Such decentralized and ecologically friendly solutions not only lower carbon footprints but also enable scalable and site-specific fertilizer generation, which is critical for sustainable food production in a changing climate.

## Author contributions

X. S., C. B., and R. N. Z. conceptualized the research idea about the interfacial nitrogen cycle. X. S. and C. B. conducted methodology development. X. S., C. B., and J. X. constructed the prototype. X. S. collected the nitrate production data by using mass spectrometry. C. B. collected the ammonia concentration data from the UV-vis colorimetry test. J. X. collected the nitrate concentration data by ion chromatography. C. B. and R. N. Z. supervised the whole research progress. X. S. wrote the original draft. C. B. and R. N. Z. revised and finalized the draft.

## Conflicts of interest

For R. N. Z., C. B., and X. S., a patent disclosure has been made to the Stanford Office of Technology Licensing: expedited chemical reactions at curved microscale interfaces between water and a hydrophobic medium. PCT application number PCT/US2024/012974 filed 25 January 2024. The other authors declare that they have no competing interests.

## Supplementary Material

SC-OLF-D5SC05754J-s001

## Data Availability

All data needed to evaluate the conclusions in the paper are present in the paper and/or the supplementary information (SI). Supplementary information is available. See DOI: https://doi.org/10.1039/d5sc05754j.
