# Supplementary material for: The air–water interfacial nitrogen cycle produces irrigatable-level ammonium nitrate
Source: Chem Sci. 2025 Oct 14;16(46):21815–24. doi: 10.1039/d5sc05754j (PMC12536449; doi:10.1039/d5sc05754j)
Supplement: SC-016-D5SC05754J-s001 [file SC-016-D5SC05754J-s001.pdf]

Supplementary Information (SI) for Chemical Science.
This journal is © The Royal Society of Chemistry 2025

SUPPLEMENTARY INFORMATION

Air-Water Interfacial Nitrogen Cycle Produces Irrigatable-Level Ammonium Nitrate

Xiaowei Song[1], Chanbasha Basheer[2]*, Jinheng Xu[1], Richard N. Zare[1]*

1. Department of Chemistry, Stanford University, CA, 94305, USA
2. Chemistry Department, King Fahd University of Petroleum and Minerals, Dhahran, 31261, Saudi Arabia
*Corresponding authors: cbasheer@kfupm.edu.sa; zare@stanford.edu

Table of Contents

Item	Caption/Heading	Page
Experimental Methods	Reagent and materials	S3
	Preparation of water-soluble photocatalysts	S3
	Preparation for the nitrate reduction catalyst-coated mesh	S3
	Nozzle spray setup for the online MS detection	S4
	Recirculating microbubble reactor for nitrate production	S4
	Continuous-spraying-recycling system for ammonia production	S5
	Nitrate quantitation by mass spectrometry and ion chromatography	S5
	Ammonia identification	S5
	Colorimetric quantitation of ammonia	S6
	XRD measurement	S6
	DFT calculation	S6

Figure S1	Diagram of three iron-anchored molecular photocatalyst synthesis	S8
Figure S2	The H_2O_2 measurement and its diffusion equilibrium between ROS enhancer unit and nitrogen oxidation reaction (NOR) unit in the microbubble system	S9
Figure S3	Characterization of the crystal surface indices of the ferrous oxide coated on the copper oxide mesh by X-ray diffraction	S10
Figure S4	Investigation of the pH impact on the nitrate reduction into ammonia by spraying nitrate-containing water microdroplets through the catalyst mesh.	S11
Figure S5	Stability test results for the Fe_3O_4-Nafion-CuO mesh	
Table S1	Consumed solar energy calculation	S12
Table S2	Consumed electric energy calculation	S12
Table S3	Consumed kinetic energy calculation	S12
Table S4	Estimated prices of the produced nitric acid and ammonia	S13

Experimental Methods

Reagent and materials

Deionized, ultrafiltered water was purchased from Fisher Chemical (W2-4, LOT 242077) for generating microdroplets and microbubbles. The compressed air (Prospec UN1002, Ultra Zero Grade) was ordered from Praxair Inc. (Danbury, CT, USA). The colorimetric test kit was commercially available in the market to check the ammonia concentration (item model number: LR8600, Mars Fishcare North America Inc., Chalfont, PA, USA). The ammonium hydroxide solution was ordered from Fisher Chemical (30% w/v, ACS grade) for quantitation purposes. Ferrosoferric oxide (black powder, Fe_3O_4) was from Spectrum Chemical MFG Corporation (769 Jersey Ave, New Brunswick, NJ, USA). The Nafion solution (5 wt%) was obtained from Fuel Cell Earth (10 Draper St, Unit 32, Woburn, MA, USA). Copper supporting foam was ordered from TMAXCN (Xiamen, China). Three photocatalysts, namely, protoporphyrin IX, 5,10,15,20-tetra(4-pyridyl)-21H,23H-porphine, and 5,10,15,20-tetrakis(4-aminophenyl)-21H,23H-porphine, were ordered from Sigma-Aldrich (St. Louis, Missouri, USA). The nitric acid and sodium nitrate were ordered from Thermo Fisher Scientific (Waltham, Massachusetts, USA). The solar concentrator (a 300 mm large optical PMMA Plastic Solar Fresnel lens) was purchased from Knight Optical UK Ltd, Kent, UK. The H-cell electrochemical reactor was provided by MSE supplier (Tucson, AZ, USA). The polytetrafluoroethylene (PTFE) powder was ordered from Sigma-Aldrich (mean particle size: 20 μm, weight: 100 g) for the contact-electro-catalyzed generation of hydroxyl peroxide under ultrasonication. The PTFE Membrane Disc Filter (C0000424, Hydrophilic, 47mm, Micron Pore Size 0.22 μm, PEKYBIO, China) was used to block the PTFE particles from the microbubble system. The diaphragm pump was provided by Huiling (HL-4A16, DC 12V, 1.2 GPM, 100 psi, China). The portable power station was used to support the outdoor experiments (Grecell T300, 288Wh, Shenzhen Intelligent Energy Co., Ltd, China).

Preparation of water-soluble photocatalysts

To prepare three water-soluble photocatalysts (PC1, PC2, PC3), An 100 mg of $FeCl_2 \cdot 4H_2O$ (M.W.198.8 g/mol) was mixed with either 280 mg protoporphyrin IX (562.7 g/mol), 305 mg 5,10,15,20-tetra(4-pyridyl)-21H,23H-porphine (618.7 g/mol), or 337 mg 5,10,15,20-tetrakis(4-aminophenyl)-21H,23H-porphine (674.8 g/mol) in the 50 mL N, N-dimethyl formamide (DMF), respectively. Three mixture solutions were stirred at 800 rpm at 50 ℃ for 6 hours. The DMF solvent was removed by rotary evaporation under low vacuum. The purple solid was purified by silica gel column chromatography.

Preparation for the nitrate reduction catalyst-coated mesh

A sample of 4 mg Fe_3O_4 was spiked into 1 mL water solution containing 0.2% Nafion (5% × 40 μL ethanol). Then, the 1 mL solution was ultrasonicated for 2 hours to prepare

catalyst dispersion ink. The dispersion ink was dropcast onto a copper oxide mesh supporter (loading area, 1.0 × 1.0 cm^2; thickness, 1.6 mm; TMAXCN, Xiamen, China) mounted on a heating plate at 145°C for complete drying. Thereafter, the prepared catalyst mesh was placed in the autoclave for baking at 300°C.

Nozzle spray setup for the online MS detection

A low-resolution linear ion trap mass spectrometer (LTQ-MS; Thermo Fisher Scientific, San Jose, CA, USA) and high-resolution mass spectrometer (Orbitrap Elite; Thermo Fisher Scientific, San Jose, CA, USA) were employed for online monitoring of nitrogen reduction to ammonia (LTQ-MS) and the nitrate reduction formation (Obitrap Elite), respectively. For ammonia detection, the LTQ-MS capillary voltage was set to −40 V, while the lens voltage was set to −20 V. The scan range was set to the "low" mode, with an *m/z* range of 30 to 60. The maximum acquisition time was 400 ms. The capillary temperature was set at 250°C. The generation of ammonia and nitrate was characterized by *m*/*z* 36 ($[NH_4OH + H]^+$) under positive mode and *m*/*z* 62 (NO_3^-) under negative mode, respectively. For the characterization of nitrate stepwise reduction, high-resolution mass spectra were acquired to capture critical nitrous oxide species associated with the nitrogen valence change during the stepwise hydrogenation process. The critical parameters were the same as those in the LTQ-MS setup, except that the scan range was set to *m/z* 50-100 under negative scan mode.

A nozzle spray setup consisted of a silica capillary (ID, 100 μm; and OD, 250 μm; Polymicro Technologies, Phoenix, AZ, USA), a syringe pump (Masterflex, Cole Parmer, Vernon Hills, IL, USA), gas cylinder, stainless steel tubing [inner diameter (ID), 500 μm; outer diameter (OD), 2 mm; and length, 30 mm], stainless-steel tee union (Swagelok, Solon, OH, USA). The nozzle spray setup was fixed in front of the LTQ-MS inlet with a 16 mm distance. A catalyst-coated mesh was mounted in the middle of the nozzle sprayer outlet and the LTQ-MS inlet. The compressed air was used as the nebulization gas with the pressure tuned to 120 psi. The flow rate was set at 10 μL/min.

Recirculating microbubble reactors for nitrate production

The nitrate production device mainly consisted of several critical functional units: (1) microbubble generation unit, which includes Teflon tubings, microbubble generator probe (YLEC consultants fluid mechanics, Mode: CARMIN D1 single, France), a water circulating pump, and an H-cell reaction chamber. This unit is used to fully disperse the air into bulk water to form microbubbles and increase the contacting area and chances between dinitrogen molecules and the air-water interface; (2) reactive oxygen species (ROS) enhancement unit, which contains water and dispersed PTFE nanoparticles to produce additional H_2O_2 to be activated into hydroxyl radicals by coupling with the iron-based porphyrin photocatalyst. (3) nitrogen oxidation unit, which was interconnected with both the microbubble generation unit and the ROS enhancement unit by Teflon tubings.

Generated microbubbles will be transferred into this unit to finish the nitrogen oxidation reaction. Meanwhile, the extra H_2O_2 generated from the ROS enhancement unit will also be diffused into this unit, whereas the 0.22 μm PTFE membrane disc filter will block the PTFE nanoparticles.

Continuous spraying system for ammonia production

The spraying-circulating setup for scaling up the ammonia production consisted of an nozzle atomizer (spray cone angle 60°, ADG SV980, China), a cylinder chamber (ID, 100 mm; and height, 200 mm), a catalyst container (ID, 25.5 mm; and height, 20 mm), a peristaltic pump (flow rate, 0 to 100 mL/min; and tube ID, 2 mm; Gikfun, China), a gas cylinder (Praxair, San Jose, CA, USA), and Teflon tubing (ID, 2 mm; and length, 300 mm). The nozzle atomizer sprays the nitrate-containing bulk water into micron-sized droplets. The cylinder chamber is made of borosilicate glass and used as a reactor. The catalyst mesh is mounted 100 mm from the bottom of the cylinder chamber. A peristaltic pump is used to transfer condensed water droplets at the bottom of the chamber back to the nozzle for water microdroplet regeneration. The water flow rate was kept consistent at 20 mL/min. The gas cylinder provides compressed air (Linde, UK) as both the nebulizing gas and the nitrogen feeding source, with the pressure set at 120 psi.

Hydroxyl peroxide measurement

Horseradish peroxidase (HRP, 0.2 mg/mL) and 3,3′,5,5′-tetramethylbenzidine (TMB, 1 mg/mL) were employed as chromogenic substrates to evaluate the hydrogen peroxide (H_2O_2) diffusion equilibrium process. After the PTFE nanoparticles dispersion in the ROS enhancer unit was ultrasonicated for 1 hour, the water sample from its connected nitrogen oxidation unit was harvested at the following time points: 0, 5, 10, 20, 30, 45, 60, 90, 120, and 180 minutes for measurement of H_2O_2 concentration. In the presence of H_2O_2, HRP catalyzes the oxidation of H_2O_2 into OH•, initiating the oxidation of TMB and causing the solution to turn a light green color.

Nitrate quantitation by mass spectrometry and ion chromatography

The concentration of nitrate anion (NO_3^-) was measured by an ion chromatograph (Dionex ICS 6000, ThermoFisher Scientific, San Jose, CA, USA) equipped with a high-capacity anion-exchange column (Dionex IonPac AS11-HC, Thermo Fisher Scientific, San Jose, CA, USA). The mobile phase was an aqueous KOH solution with a flow rate of 1 mL/min. A conductivity detector analyzed ions. Dilution series of $NaNO_3$ aqueous solutions were used as standards for the nitrate quantification in this experiment.

Ammonia identification

Ammonia formation was confirmed using the indophenol blue colorimetric method. The colorimetric test was conducted by using the commercially available test kit (item model

number: LR8600, Mars Fishcare, USA). For each 1 mL sample solution, 200 μL test kit solution A and 200 μL test kit solution B were sequentially spiked and thoroughly shaken to observe the color change after 10 minutes of reaction at room temperature. Ultraviolet-visible spectroscopy, equipped with a 96-well plate array readout system (Abbott Laboratories, Illinois, USA), was used to acquire the sample absorbance within the range of 220 to 800 nm and confirm the maximum absorbance wavelength.

Colorimetric quantitation of ammonia

Four drops of Ammonia Colorimetric Test Kit solutions (A) and solution (B) were added to 0.5 mL samples to develop the indication color after 10 minutes. A quantitation curve was constructed by testing dilution series of ammonium hydroxide standard solutions. A stock solution was first prepared at a concentration of 200 mg/L (parts per million, ppm). Then, a series of ammonia solutions (0.2, 0.5, 1.0, 2.0, 5.0, 10.0, and 20.0 ppm) were prepared by gradual dilution with HPLC-grade pure water. Three replicates of 0.5 mL sample or ammonia solution at each concentration were measured using the UV-Vis system with the colorimetric method described above. After 10 minutes of colorimetric reaction, the absorbance of the solution at the wavelength of 690 nm was used to determine concentration. The calibration curve was plotted by fitting the absorbance, corrected for background subtraction, versus concentration.

X-ray diffraction (XRD) measurement

The Fe_3O_4-Nafion-coated CuO mesh was cut into a small cube piece (5 mm × 5 mm × 2 mm) for catalytic use. The XRD spectra were collected using a Benchtop Bruker D2 Phaser Diffractometer (USA). Cu Kα excitation (λ=0.15406 nm) was employed to investigate the crystal phase of the Fe_3O_4 catalyst and CuO supporting mesh. All XRD peaks are well indexed to the tetragonal CuO and Fe_3O_4 phases, which match JCPDS cards of No. 45-0937 (for CuO) and No. 19-0629 (for Fe_3O_4). The sharp and strong diffraction peaks indicate the excellent crystalline nature of the prepared CuO. The diffraction peaks located at 2θ = 36.98°, 39.61°, and 62.05° correspond to reflections from the Cu (111), (200), and (220) crystal planes, respectively. The diffraction peaks located at 2θ = 36.98°, 39.61°, and 62.05° correspond to reflections from the CuO (002), (202), and (113) crystal planes, respectively. The diffraction peaks located at 2θ = 36.98°, 39.61°, and 62.05° correspond to reflections from the Fe_3O_4 (311), (400), and (440) crystal planes, respectively.

Density functional theory (DFT) calculation

Density functional theory (DFT) calculations were performed using the Dmol3 module of Material Studio 2020. The generalized gradient approximation (GGA) method, with the Perdew-Burke-Ernzerhof (PBE) functional, was employed to describe the interactions between the core and electrons. The force and energy convergence criteria were set to

0.002 Ha Å^{-1} and 10^{-5} Ha, respectively. The surface of Fe_3O_4(311) was obtained, and 15 Å vacuum spaces were implemented into the model to eliminate undesirable interactions between the bottom side of the slab and the molecules in the vacuum space. The (2 x 2) unit cell of Fe_3O_4(311) was used to prohibit lateral interactions between molecules on the surface. When the optimization was completed, the frequency calculations were performed. Then the free energy was obtained.

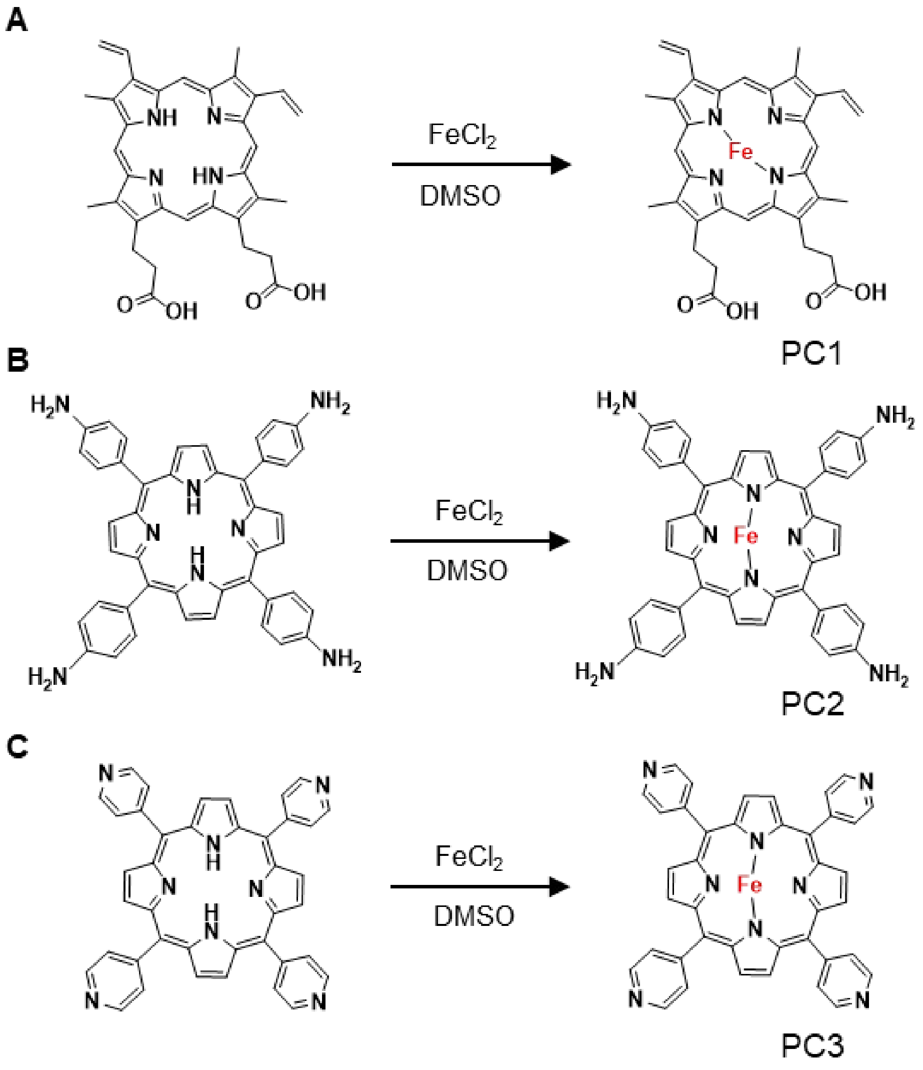

Figure S1. Diagram of three iron-anchored molecular photocatalyst synthesis.

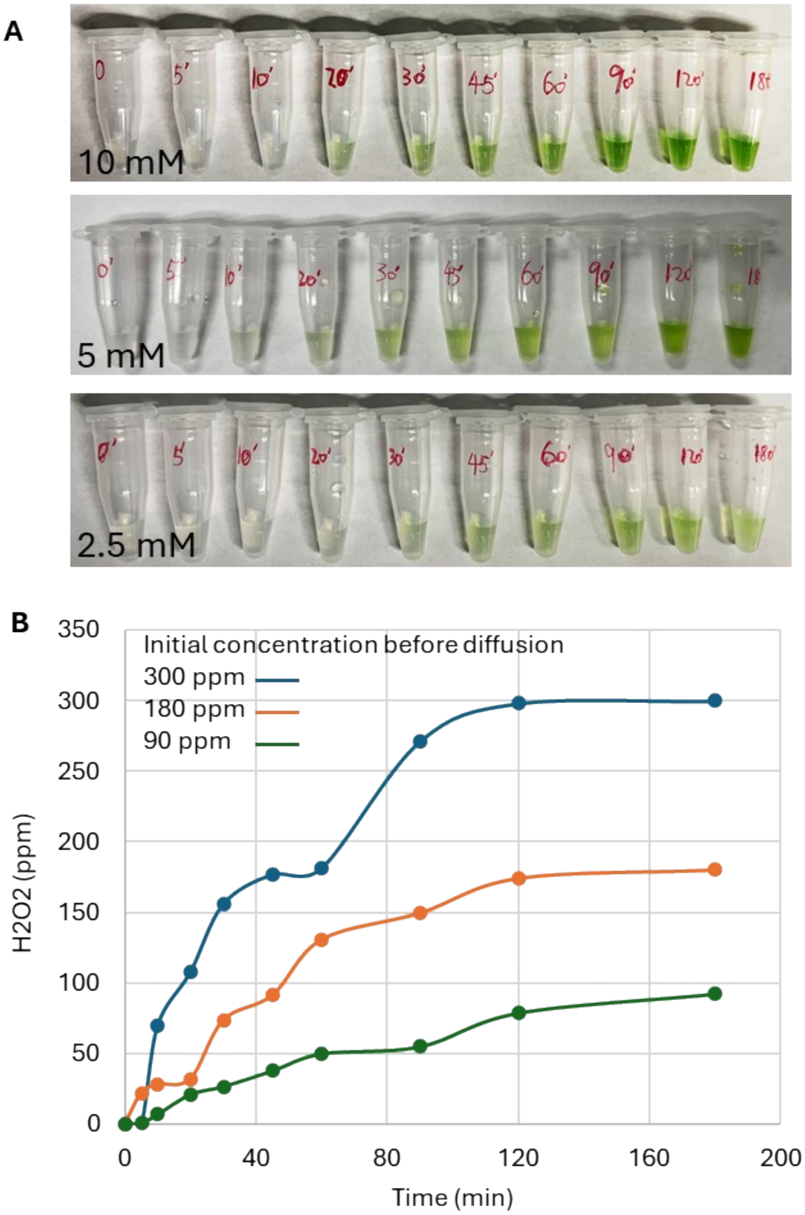

Figure S2. The H_2O_2 measurement and its diffusion equilibrium between ROS enhancer unit and nitrogen oxidation reaction (NOR) unit in the microbubble system. (A) The measurement of H_2O_2 concentration in the NOR unit. (B) Diffusion equilibrium curves of the H_2O_2 in the ROS enhancer unit at three different initial concentrations before diffusion.

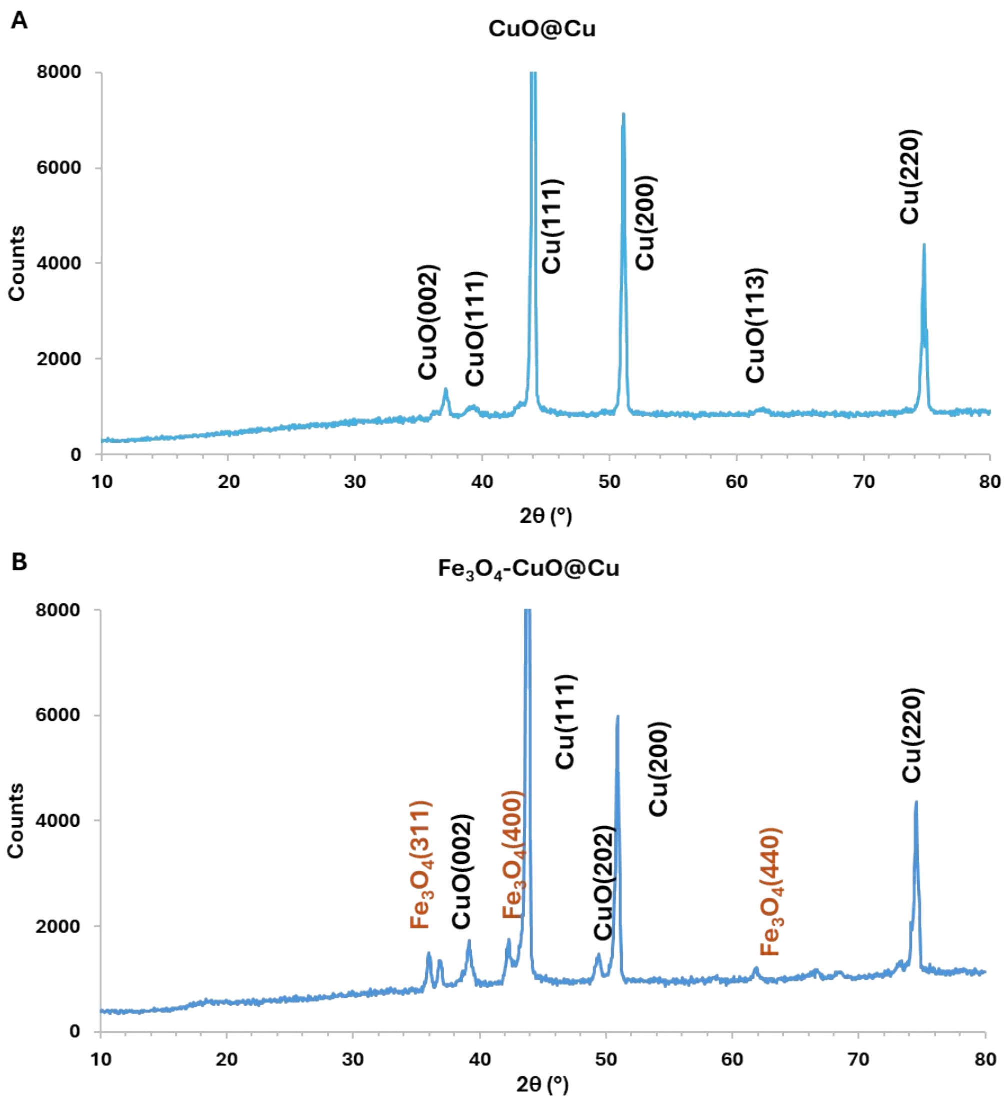

Figure S3. Characterization of the crystal surface indices of the ferrous oxide coated on the copper oxide mesh by X-ray diffraction (XRD). (A) The XRD spectrum of CuO@Cu mesh. (B) The XRD spectrum of Fe_3O_4-Nafion@CuO mesh.

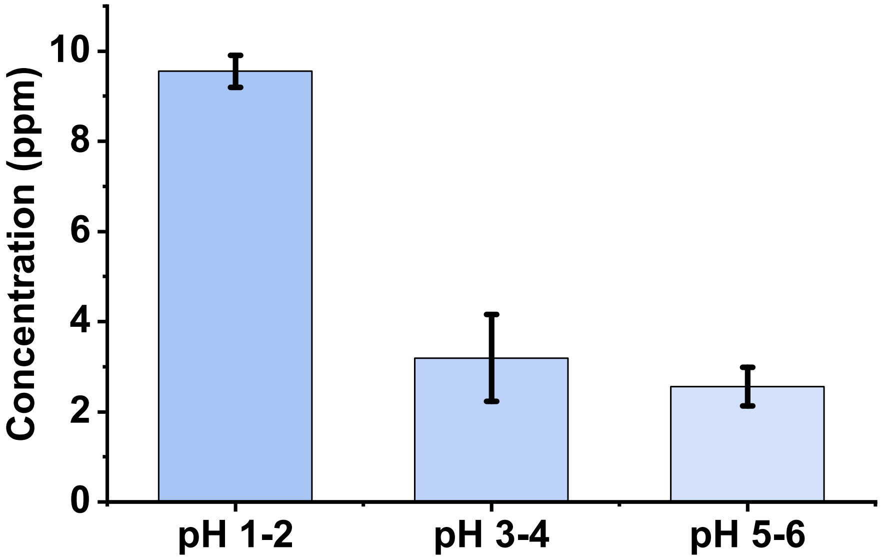

Figure S4. Investigation of the pH impact on the nitrate reduction into ammonia by spraying nitrate-containing water microdroplets through the catalyst mesh.

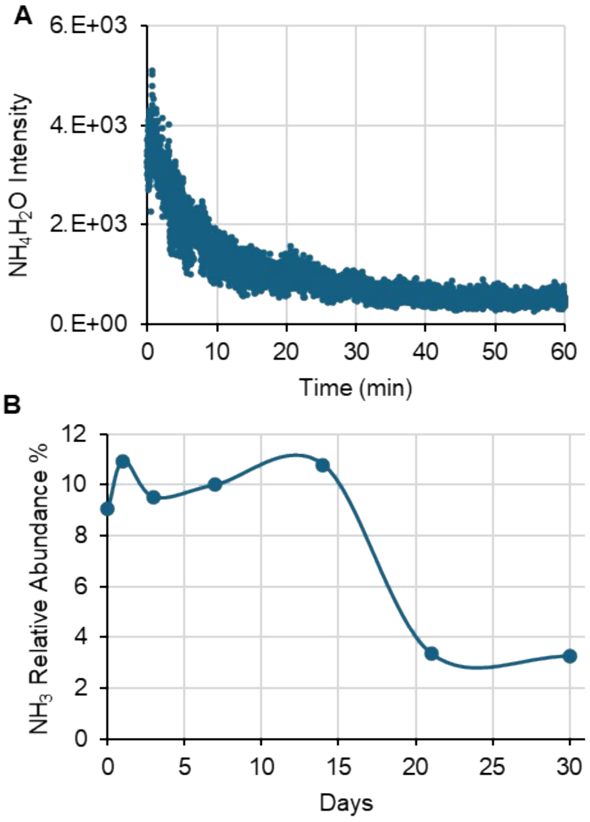

Figure S5. Duration and stability test results for the Fe3O4-Nafion-CuO mesh.

Table 1. Consumed solar energy calculation

Items	Value	Unit
average solar irradiation on earth	750	W/m^2
reactor diameter	0.1	m
irradiation area	0.00785	m^2
time	12	h
	43200	sec.
work	32400	kJ
	9.0	kWh

Table 2. Consumed electric energy calculation

Items	Value	Unit
voltage	12	V
current	3.7	A
power	44.4	W
time	13	h
work	2077.9	kJ
	0.5772	kWh
average electric fee in the U.S. market	0.17	$/kWh
price	0.10	$

Table 3. Consumed kinetic energy calculation

Items	Value	Unit
compressed gas pressure	100	psi
	689476	Pa (N/m^2)
nozzle diameter	2	mm
impact area	3.14E-06	m2
spray distance	0.3	m
gas velocity	50	m/sec
power	108.2	W
time	1	h
	3600	sec.
work	389.7	kJ
	0.11	kWh

Table 4. Estimated prices of the produced nitric acid and ammonia

Items	Value	Unit
HNO_3 concentration	5.36	mmol/L
NH_3 concentration	0.94	mmol/L
volume	50	mL
HNO_3 amount	0.000268	mol
NH_3 amount	0.000047	mol
HNO_3 weight	0.016884	g
NH_3 weight	0.000799	g
cost	0.10101	$
HNO_3 Price	5.982587	$/g
NH_3 Price	126.4205	$/g
